# The Transcriptional and Gene Regulatory Network of *Lactococcus lactis* MG1363 during Growth in Milk

**DOI:** 10.1371/journal.pone.0053085

**Published:** 2013-01-17

**Authors:** Anne de Jong, Morten E. Hansen, Oscar P. Kuipers, Mogens Kilstrup, Jan Kok

**Affiliations:** 1 Department of Molecular Genetics, University of Groningen, Groningen Biomolecular Sciences and Biotechnology Institute, Groningen, The Netherlands; 2 Center for Systems Microbiology, Institute of Systems Biology, Technical University of Denmark, Lyngby, Denmark; Memorial Sloan Kettering Cancer Center, United States of America

## Abstract

In the present study we examine the changes in the expression of genes of *Lactococcus lactis* subspecies *cremoris* MG1363 during growth in milk. To reveal which specific classes of genes (pathways, operons, regulons, COGs) are important, we performed a transcriptome time series experiment. Global analysis of gene expression over time showed that *L. lactis* adapted quickly to the environmental changes. Using upstream sequences of genes with correlated gene expression profiles, we uncovered a substantial number of putative DNA binding motifs that may be relevant for *L. lactis* fermentative growth in milk. All available novel and literature-derived data were integrated into network reconstruction building blocks, which were used to reconstruct and visualize the *L. lactis* gene regulatory network. This network enables easy mining in the chrono-transcriptomics data. A freely available website at http://milkts.molgenrug.nl gives full access to all transcriptome data, to the reconstructed network and to the individual network building blocks.

## Introduction

Dairy lactic acid bacteria [LAB] such as *Lactococcus lactis* thrive in milk, a nutritionally rich medium that can efficiently support their sugar-based fermentative lifestyle. Milk must have been adopted by LAB in the last approximately ten thousand years, because milk storage is exclusively a human cultural phenomenon. LAB are very important in the food industry for their ability to produce healthy, safe and tasteful foods with extended shelflifes. Thus, LAB are studied intensively to obtain fundamental as well as application-oriented knowledge. With respect to the former, recent years have seen the elucidation of the genomic sequences of several dairy and non-dairy LAB. Among the best studied of these is *L. lactis* strain MG1363 [1]. The genome sequences have been used for extensive (phylogenetic) comparisons. Importantly, they have allowed examining genome-wide analyses by DNA microarray technology of a number of LAB species [2], [3]. These studies and earlier work has led to the detailed description of many metabolic and regulatory networks in *L. lactis*, such as purine and pyrimidine biosynthesis [4], [5], [6], amino acid biosynthesis [7], peptide uptake and degradation [8], transcriptional regulators[9], [10] and global transcriptional regulators such as CcpA [11] and CodY [12], [13]. Many of the studies presented so far entail single time point analysis of e.g., a genetic mutation (mutant-parent analysis). It is quite a challenge to examine the behavior of a bacterium's transcriptome in time during growth in a complex medium such as cheese [14]. Here, we performed chrono-transcriptomics of *L. lactis* fermentation of milk.

Vitamins and sugar (lactose) are readily available to *L. lactis* in milk but it has to actively liberate amino acids from milk proteins (caseins) by proteolysis. It is generally believed that the multiple auxotrophies in LAB have accumulated as a consequence of the abundance of growth supplements in milk [2]. This has made bacteria dependent on the correct (temporal) release/use of all essential growth factors for optimal growth.

When *L. lactis* is growing in milk it will have to meet several challenges to survive in an ever-changing environment; changing concentrations of amino acids, peptides, sugars, (an)organic compounds, decrease of pH and increasing cell density and ultimately, nutrient limitation. Many of these changes should be visible as a response in the gene transcriptional network a large part of which will be controlled by transcriptional regulators [15], [16]. Analysis of these responses by DNA microarrays will provide insights on when and how transcriptional regulation is managed in the cell. Monitoring mRNA levels and production profiles offers a key to how gene expression is regulated in response to the changing environment. Transcription regulators affect gene expression by binding to specific upstream DNA regions. Computer algorithms [MEME [17], SCOPE [18]] can be used to mine for conserved DNA regions (DNA binding motifs) in the promoter regions of co-regulated genes. When a DNA binding motif is located in separate promoter regions, in addition to those of co-regulated genes, this indicates that these additional genes may be under the control of the same regulator [10].

To supply data for a gene regulatory network of *L. lactis* in its natural and also in the food industrial environment, we cultured two biological replicates of the *L. lactis* MG1363 in milk and performed temporal transcriptome analysis using DNA microarrays.

## Materials and Methods

### Growth conditions

Milk medium was prepared by heat-treating 10% reconstituted skimmed milk at 90°C for 30 min. The milk was inoculated with a 1/20 volume of an exponentially growing culture of *Lactococcus lactis* MG1363 carrying pLP712, a plasmid containing the genes to degrade lactose (Lac+) and proteins (Prt+) [19], in milk shortly after the temperature had reached 30°C. The inoculum of *L. lactis* MG1363 had been growing exponentially in milk at 30°C for approximately 5 generations and had reached a pH of 5.5. The skimmed milk powder was a gift from Arla Foods, Viby, Denmark.

### Determination of colony forming units and pH

Medium pH was monitored by taking samples at appropriate time points and measuring pH with an electrode. Colony forming units were determined by appropriately diluting samples in M17 and plating on M17 (Difco, USA) agar plates containing 0.5% w/v glucose. Colonies were counted after overnight incubation at 30°C.

### Total RNA extraction from milk cultures and DNA microarray methodology

Cellular processes were quickly quenched by rapid cooling of 10 ml samples in large glass flasks (with large heat capacity) pre-chilled in an ice-ethanol bath. Following centrifugation at 6000 g for 10 min at 4°C, the pellet was resuspended in 1 ml ice-cold 3 M guanidinium chloride. Following solubilisation of the milk coagulate by five cycles of vortex mixing and chilling on ice, each for one minute, cells were collected from the cleared solution by centrifugation at 4°C at 3500 g for 15 min. After two washed in ice cold 0.9% saline solution the cell samples were frozen at −80°C or used directly for RNA extraction.

RNA was extracted by the hot phenol method as described before [5]. Subsequently, cDNA was obtained and labeled with Cy3 and Cy5 dyes and slide hybridization was performed at 45°C as described previously [20] on in-house spotted *L. lactis MG1363* DNA microarrays (for details see platform GPL5048 in the GEO database [21]).

### DNA microarray data normalization

Intensity levels of the signals on the slides in the different data sets were scaled to equal total intensities and normalized with lowess grid-based normalization using Microprep [22]. This scaling is equivalent to assuming that the total mRNA concentration in the cell is constant throughout the experiment; although this assumption is not true for absolute levels it is valid for the relative expression profiles of genes. The raw data and the normalized data are deposited in Base2 [23]. Global analyses were performed on these scaled normalized intensity level data. The sets of intensity values were normalized and combined into (I) intensity ratios, corresponding to ratios between mRNA levels from two time points; and (II) intensity levels, corresponding to absolute mRNA levels for each time point.

### DNA microarray data analysis

Genome2D [24] was used to visualize expression profiles and to calculate the hypergeometrical distribution of classes. TM4 [25] was used for Principle Component Analysis (PCA). The method of Ahdesmaki et al. [26] was used to filter out genes with very low expression levels in order to circumvent false correlation of background signals in GeneNet. To find links between operons with (partial) co-expression, GeneNet [27] was used to calculate gene interactions. To calculate the Pearson's correlation between gene expression profiles an *in-house* routine in R [28] was developed (available on request). The sliding window routine is a modified R routine based on GeneCycle [29] (available on request). DISCLOSE [30] was used to mine for conserved DNA motifs. Cytoscape [31] was used to reconstruct and visualize the gene network.

### Genome annotation data

The genome annotation data of *L. lactis* MG1363, AM406671_GR, was retrieved from the Genome Reviews database of EMBL-EBI (http://www.ebi.ac.uk/GenomeReviews/). This Genome Review file was used as a source for the COG classification. In total 102 pathways were extracted from the metabolic data base of KEGG (http://www.genome.jp/kegg/) using the organism-specific entry ‘llm’ of MG1363.

### Operon prediction

Little or no information is available on operons in bacterial genomes. Several tools for the *in silico* examination of operons are available but none gives a reliable operon prediction [32]. Here we used TransTermHP [33] to predict transcriptional terminators. Subsequently, Genome2D ([24] was used to generate putative operons based on the results of TransTermHP (see Supplementary Materials).

### DNA motif mining

Natural log-transformed gene expression ratio data from this milk fermentation time series was used as a data source to mine for (novel) transcription factor binding sites (TFBS) in the genome of *L. lactis* MG1363. COG classification and GO annotation of the genes of *L. lactis* MG1363 were derived from NCBI and EBI, respectively. The PePPER database [34] was used as a source of literature-based regulon classes. These data sources were loaded in DISCLOSE [30] followed by *k*-means clustering using Pearson, Spearman and Euclidean distance settings on 10 to 60 clusters. All clustering settings were tested for overrepresentation of classes (pathway, GO, COG and regulon) within one cluster. The sum of overrepresented classes in each cluster represents the total score of a clustering setting.

We combined DNA-motif mining with various gene classifications (operon, GO (http://www.geneontology.org/) and metabolic pathways (http://www.genome.jp/kegg/)) and subsequently validated the obtained DNA-motifs with literature data. A DNA-motif was assigned valid if electrophoretic mobility shift assays (EMSA's) or DNA foot printing data were available in the primary literature describing the motif. For validation on the basis of classes, a weight matrix was made using the DNA-motifs obtained by DISCLOSE, followed by a genome-wide screen using this weight matrix. A hypergeometrical test was performed on the obtained sequences to screen for DNA motif-enriched classes. If an enriched class was found, the DNA-motif was marked as valid.

### Software and webserver

For calculation of gene-to-gene correlation over time we used GeneNet version 1.2.4 (http://strimmerlab.org/software/genenet/), which is an R package (http://www.r-project.org/) for obtaining high-dimensional dependency networks from genomic data [27]. Cytoscape 2.7.0 [31] was used for building and visualizing of the gene regulatory network (http://www.cytoscape.org/). Genome2D [24] was used for motif visualization and mining in genomic data. The interactive website which contains the resource data described in this article (http://milkts.molgenrug.nl) was built in Joomla using PHP for session management and R for graphical presentation of the data.

### Reconstruction of the Gene Regulatory Network

An interactive Gene Regulatory Network (GRN) was built using all data of the analyses described in this paper combined with literature data. To this end, distinct data sets were made that describe relation/correlation between genes, transcription profiles, operons, regulons, TFBSs and metabolic compounds. The *first* relation between genes is the correlation (Pearson's correlation) of their expression profiles over the complete time-series. The *second* relation is based on the results of the time sliding analysis, which describes when the expression of a gene changes significantly during growth. For the *third* correlation, a *p-*value describing the putative interactions between genes was calculated using the network reconstruction algorithm GeneNet [25]. The *fourth* relation between genes is based on the fact that genes belonging to one operon are transcribed from one mRNA and, therefore, will show the same or very similar expression profiles. This relation was established by our operon prediction method, which does not completely match the real situation because operon prediction algorithms do not predict mRNA stability or dynamic changes in mRNA levels [34]. The *fifth* relation is based on shared DNA motifs in upstream regions of genes or operons, indicating that they may be controlled by the same transcriptional regulator. This correlation is based on literature-derived DNA motifs supplemented with novel motifs discovered by DISCLOSE [30] using the time-series data and functional annotation of genes. The *sixth* relation is based on regulon data derived from literature and on our *in-house* regulon data sets. The *seventh* relation is between genes and compounds; it is based on the metabolic reaction database specific for *L. lactis* MG1363 [35], [36] that couples production and consumption of compounds to gene products. The results of all seven correlation analyses were stored in a Cytoscape [31] compatible format and include their weight factors, if applicable. Cytoscape “Merge Networks” and the Cytoscape “plug-in” CABIN [37] were used to combine the seven data sources to create a gene regulatory network.

## Results and Discussion

### Standardization of milk fermentation conditions

Milk is a very complex medium that changes continuously during fermentation by *L. lactis* as the milk protein (casein) is being degraded by the bacterial proteases and the resulting peptides and amino acids, as well as vitamins, are used. At the same time, lactose is being consumed and lactate and other fermentation products are being produced, changing the pH of the medium. An outgrown frozen or freeze-dried milk culture is typically used to inoculate fresh milk for fermentations. Because such cultures are acidic and the bacteria have undergone an acid stress response, their physiological status is highly unpredictable. To ensure the highest degree of reproducibility in this study, the milk medium was inoculated with an exponentially growing culture of *L. lactis* in milk. Even with these precautions it should be noted that milk will never support balanced growth of any bacterium. Balanced growth is the only state with a defined physiological status of the bacterium, so not even theoretically can full reproducibility be attained with a milk medium.

**Figure 1:**
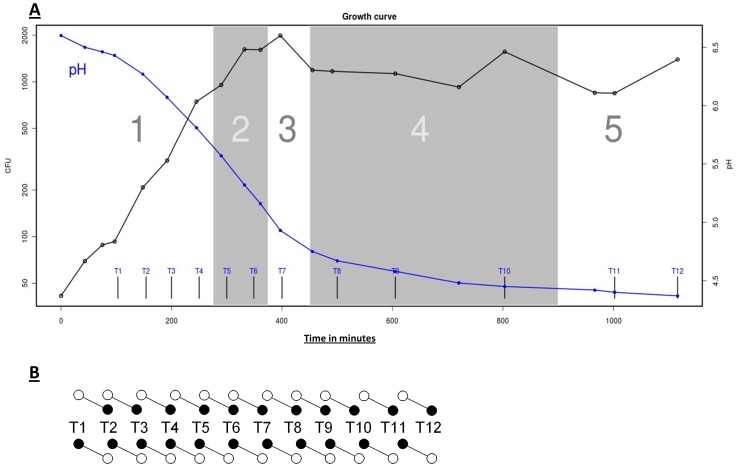
*L. lactis* MG1363 chrono-transcriptomics set up. **A**) Growth of and acidification by *L. lactis* MG1363 in milk. Cell concentration in colony forming units (CFU) per ml and medium pH are shown on the left and right axis, respectively. Vertical lines indicate the time points of sampling (T1 to T12). The major five phases of gene transcription, based on PCA in Fig. 2 are marked in white and grey boxes numbered from 1 to 5. **B**) Fluorescently labeled cDNA samples (T1–T12) were mixed according to scheme shown; e.g., Cy-5 labeled (filled circle) sample T1 was combined with Cy-3 labeled (open circle) sample T2.

The growth rates (as measured by the number of colony forming units in time) and lactate production (as measured by medium pH) are in close accordance for the two parallel cultures, A and B (Fig. 1A; data only shown for culture A). Both growth curves are characterized by at least two distinct phases, an exponential phase between 100 and 400 min after inoculation and a stationary phase after 400 min, which coincides with a drop in pH below 5.0. Entry into early stationary phase can be recognized after 250 min by a slight decrease in the growth rate at the point where oligopeptides present in the sterilized milk become limiting [38]. At the time points indicated with arrows in Fig. 1A, samples were withdrawn from each culture and used for transcriptome analysis.

### Genome-wide transcription analysis

At the end of the fermentation, the milk medium had fully coagulated but our novel extraction technique, using 3 M guanidinium hydrochloride (the procedure will be published separately), enabled us to obtain pure bacterial fractions from these late stages of growth. From each of the two milk fermentations, RNA samples were obtained over the full fermentation period, from 100 min to 1100 min after inoculation. In this way, a total of 12 samples were taken from each culture (T1–T12). The RNA was analyzed by DNA microarray analysis according to the scheme shown in Fig. 1B. Intensive data analysis was done on the results derived from the fermentation culture A while the second data set (derived from culture B) was used to verify and/or confirm observations in the data set of culture A. The DNA microarray raw data sets and normalized ratio data can be downloaded from the NCBI GEO database [19] as series GSE40780 and scaled signal data can be found at the accompanying website to this paper (http://milkts.molgenrug.nl).

### A global view of the transcriptome of *L. lactis* MG1363 during milk fermentation reveals temporal gene expression responses

The analysis of the transcriptome data from culture A followed a two-step procedure. First, the data was divided into distinct response periods using algorithms for the mining for global trends. Second, each of these response periods was then characterized by identifying the Clusters of Orthologous Groups (COG) [39] that responded most significantly.

**Figure 2:**
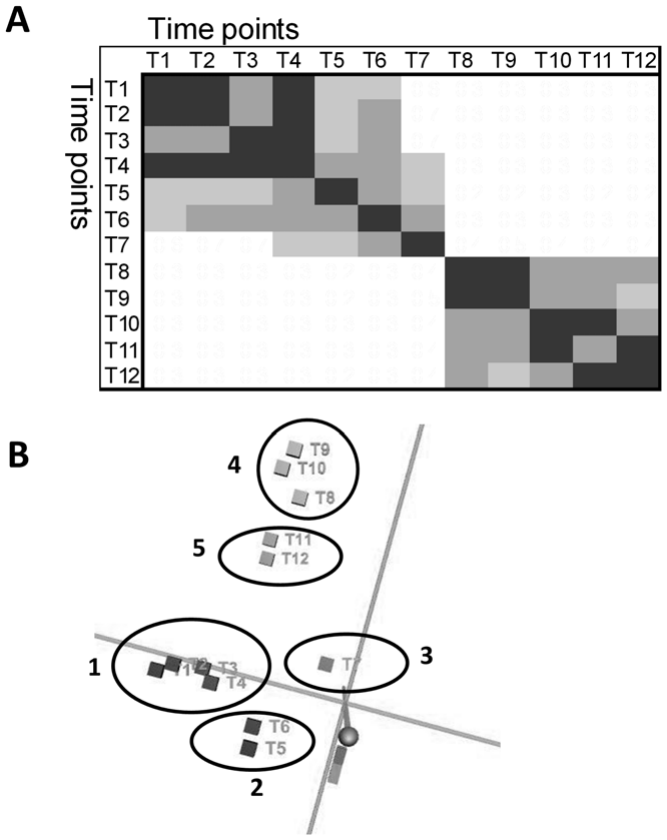
Correlation between time points. **A**) Pearson's correlation. Sets of intensity levels from each time point (average of the two biological replicates) were analysed as separate curves with shapes determined by the intensity levels of the genes as a function of the gene number. Pearson's correlation was calculated for pairs of these genome-wide intensity curves and are shown in the T1–T12×T1–T12 matrix. Grey scale: decreasing intensity corresponds to decreasing correlation; white: no correlation. **B**) PCA. Projection of the 12 time points using 3 principle components shows 5 distinct clusters; 1: T1–T4, 2: T5–T6, 3: T7, 4: T8–T10 and 5: T11–T12.

A principal component analysis (PCA) of the intensity levels showed a division of the time points into three distinct response periods (Fig. 2B). Time points T1–T6 cluster together as do time points T8–T12 while time point T7 assumes an intermediate position. The first cluster could be argued to consist of a time point T1–T4 sub-cluster and a time point T5–T6 sub-cluster. A homogenous trajectory was manually drawn from sub-cluster T1–T4 through sub- cluster T5–T6 and time point T7 to cluster T8–T12, suggesting that the changes in gene expression are gradual.

A somewhat similar grouping of the data set was obtained when a Pearson's correlation was calculated between all time points (Fig. 2). Highest correlation of gene expression exists between time points T1 to T7 and between time points T8 to T12, whereas no correlation was observed between these two major clusters. Within the T1–T7 cluster, the correlation was strongest between T1, T2, T3 and T4 and somewhat less between T5 and T6.

Together, the PCA and Pearson's correlation analysis suggest a division of *L. lactis* gene expression during milk fermentation into five distinct temporal response groups. The characteristics of each response period are shown in Table 1.

**Table 1:** Response periods identified for *L. lactis* during fermentation of milk.

Temporal response group	Time point	Time after innocculation (min)	Cell density×10^8^ ml^−1^	pH range	Number of upregulated genes	Number of downregulated genes	Responding COG#
**Early exponential**	T1, T2, T3, T4	100–250	1–6	6.7 – 6.1	40–50	20–40	C, E, F, L, O, P, T
**Late exponential**	T5, T6	300–350	8–11	5.9 – 5.2	50–70	30–60	C, D, E, F, O
**Early stationary**	T7	400	12	5.1	130	180	C, J, O, S, T
**Middle stationary**	T8, T9, T10	500–800	10	4.6 - 4.5	50–140	20–220	C, O, P, S, T
**Late stationary**	T11, T12	900–1100	10	4.5 – 4.3	0–30	0–50	

# Letters correspond to the standard COG abbreviation.

The fact that the fermentation process can be divided into response periods with respect to gene expression levels implies that the cells encountered different stressful conditions at four critical points in time, namely at T4, T7, T9, and T10. The nature of the stress conditions is so far unknown, although the increasing acidity and the accumulation of increasingly more lactate in the medium must be important in this respect. The response of the different gene clusters suggests certain changes in metabolite concentrations and intracellular environment.

To proceed from the analysis of overall global trends in gene expression towards trends in expression of individual genes, the number of genes with significantly high intensity ratios throughout the fermentation was assessed i.e., the genes of which the expression level changed significantly between two consecutive time points (Table 1). A minor response period was seen from time point T4 to T6, within the exponential growth phase. In this period, the expression levels of approximately 40 to 80 genes significantly changed between consecutive time points (data not shown). At the very end of the exponential growth phase (T6 and T7), when the culture reaches a pH of 5.0, a high number of genes (more than 200) showed a significant change in expression. The analysis shows that a marked response takes place when the culture enters the stationary phase, at T7. The expression levels of approximately 230 genes changed significantly in the period from T7 to T8. In the next period (T8–T10), characterized by a 1.7-fold reduction in the number of colony-forming units, the expression levels of approximately 40 to 50 genes changed significantly between consecutive time points. Finally, from time point T10 up to time point T12, the expression of only a few genes (4 to 12) changed.

### Responses of functional gene classes

**Figure 3:**
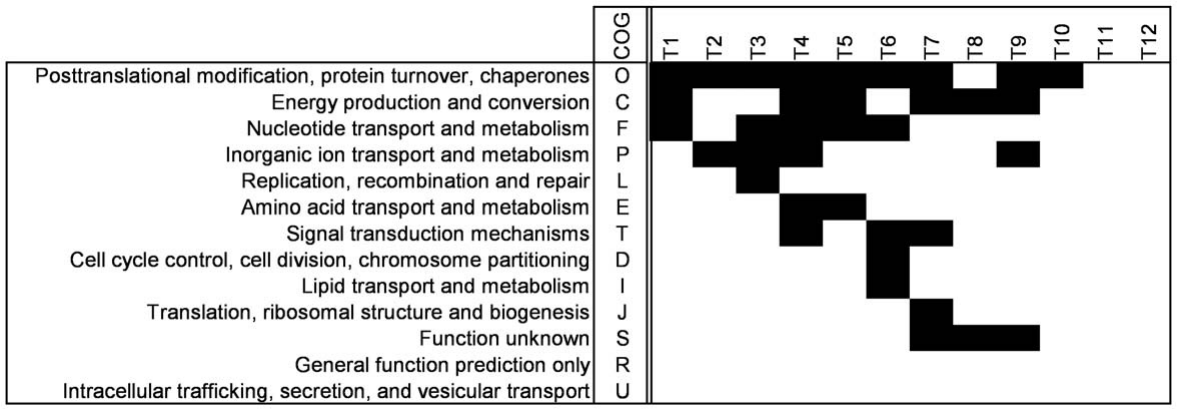
Overrepresentation of COG classes. For all time points indicated on the horizontal axis a hypergeometrical test was performed to find the overrepresented classes; the black squares indicate a significant change. Only COG classes that are significantly changed at least at one time point are shown.

A hypergeometrical test was performed to calculate the *p*-values for the change of expression of the genes in all COG classes. Fig. 3 shows in which period the major COG classes changed significantly during the fermentation. It is clear that the early exponential phase, from T1 to T4, is not a balanced state because if it were, no orthologous classes would have shown up. It is not even a stable period, as can be seen from the response within the COG of “*Nucleotide Transport and Metabolism*” time points T1–T4. This group of genes actually shows differential responses at all time points, except during exponential growth (T2–T3) but after T7 this COG class is pacified. Interestingly, genes involved in “*Inorganic ion Transport and Metabolism*” respond from time point T2 to T5, and again in the mid-stationary phase (T9–T10). Entry into the late exponential phase (T3–T4), is specified first by a response of genes of the COG “*Replication, Recombination and Repair*”, and later by a response of genes involved in “*Signal Transduction*” (T4). Throughout the late exponential phase (T4 to T6) there is a response of genes involved in “*Amino Acid Transport and Metabolism*”, suggesting that the general availability of amino acids gradually changes in this growth phase. This COG responds concomitantly with genes involved in “*Energy Production and Conversion*” during the exponential growth phase, but not so in the stationary phase.

Upon entry into the early stationary phase, from T6 to T7, a second response of the COG class “Signal Transduction” is observed. A new group of genes involved in “*Translation, Ribosomal Structure and Biogenesis*” responds for at the transition point from exponential to stationary growth (T7–T8). Throughout the early stationary phase (T7 to T9), the group of “Unknown” genes responds. A significant response from genes involved in “*Posttranslational Modification, Protein Turnover and Chaperones*” is detected between all consecutive time points, except for time point T8–T9. This may suggest a change in protein turnover as many of these genes are regulated by the heat shock response [40].

**Figure 4:**
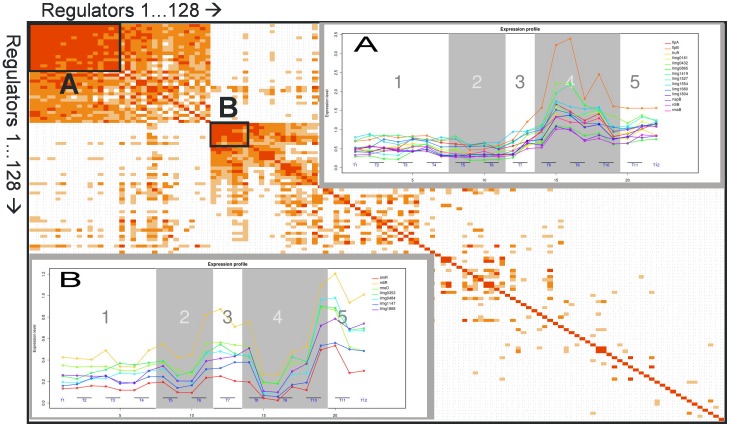
Correlation between temporal expression patterns for all (putative) *L. lactis* regulator genes. The Pearson's correlation coefficient from pair-wise comparison of expression levels of all (putative) regulator encoding genes of *L. lactis* MG1363 (http://pepper.molgenrug.nl [34]). An increase in redness indicates an increase in gene to gene correlation; white: no correlation. Box A and B are clusters of genes with high internal correlation, the temporal gene expression patterns of which are shown in the insets A and B, respectively. Transcriptional regulators in box A; *flpA*, *flpB*, *fruR*, *llmg0161*, *llmg0432*, *llmg0865*, *llmg1419*, *llmg1527*, *llmg1554*, *llmg1660*, *llmg1804*, *llrC*, *napB*, *rdrB*, and *rmaB*. Transcriptional regulators in box B; *arsR, mtlR, rmeD, llmg0353, llmg0484, llmg1147, llmg1868*.

A customized functional group of genes, namely the 128 putative and the known transcriptional regulators [41] in the genome of *L. lactis* MG1363 was analyzed with respect to possible gene-to-gene expression correlations. In Fig. 4, rows and columns are shown with descending probabilities, emphasizing the high correlation that exists between the members of the first cluster, consisting of 15 regulator genes (Fig. 4, box A), and between the 6 genes of an unrelated second cluster (Fig. 4, box B). Whereas box A genes show a constant expression level before T7 followed by an induction phase (Fig. 4, inset A), the second group of genes shows a more complex behavior. Both expression patterns reveal the presence of a critical point around T7, while for box B genes a second imported moment in time is after T9.

The analyses presented above show that at defined stages during growth, cells have gene expression transition points, which are probably caused by adaptation of the cells to a changing environment (pH, carbon source, nutrients). More in-depth data mining was done on metabolic pathways that change significantly at the transition points. First, the set of genes that changed more than 1.8 fold with Cyber-T *p*-values<10^−3^ was used to calculate, for each two consecutive time points, which KEGG pathways were significantly changed on the basis of a hypergeometrical test (Supplementary Table S1). Analysis of purine and pyrimidine metabolism was done in more detail because it plays a central role in cell growth. Second, sugar and amino acid limitation effects were studied by analysis of sugar transport and arginine metabolism. To reveal whether the cells undergo stress at these gene expression transition points, an analysis of the stress response genes was also performed.

### Expression profiles of genes involved in nucleotide metabolism define changes in PRPP and nucleotide availability

**Figure 5:**
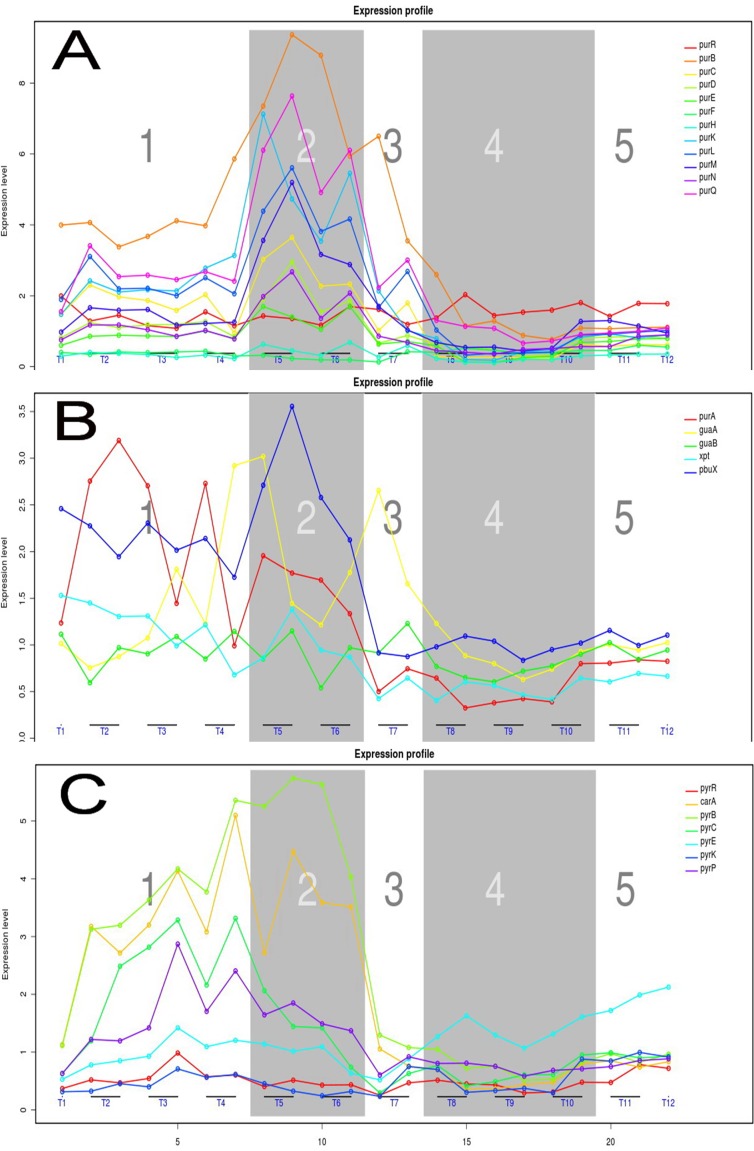
Transcription profiles of purine and pyrimidine metabolism genes. **A**) PurR regulon members; *purB*, *purC*, *purD*, *purE*, *purF*, *purH*, *purK*, *purL purM*, *purN*, *purQ* and *purR* (red line). **B**) Transcription profile of; *purA*, *guaA*, *guaB*, *xpt* and *pbuX*. **C**) PyrR regulon members; *carA*, *pyrB*, *pyrC*, *pyrDb*, *pyrE*, *pyrK*, *pyrP* and *pyrR* (red line).

Purine and pyrimidine nucleotides, the building blocks for DNA and RNA synthesis, are formed *de novo* from 5-phosphoribosyl-1-pyrophosphate (PRPP) and amino acids in *L. lactis* and most other bacteria [5]. Through alternative salvage pathways, nucleotides can be synthesized from PRPP and either nucleosides or nucleobases. Most of the purine *de novo* genes are regulated by the PurR activator, which responds in a feed-forward manner to the concentration of PRPP. The expression patterns of the known PurR regulon members in the nucleotide orthologous group clearly show that at least two critical time points exist before T7 (Fig. 5A). From T5 to T9, the expression of the PurR regulon members drops, indicating that the amount of PRPP had decreased from high at T5 to low at T9. In contrast, the sharp rise in PurR regulon gene expression from T4 to T5 indicates that PRPP accumulated from an intermediary level at T4 to a high level at T5. A short period of decreasing PurR regulon mRNA from T2 to T3 is observed for almost all PurR regulon members. The PurR regulon is a good example of the lack in coordination between the expression of regulator and target genes, as the temporal expression curve of the *purR* gene (red line in Fig. 5A) is almost flat. The *purR* gene is known to be under PRPP-independent repression control by binding of PurR to its promoter region. In contrast, the PurR regulon members are under PRPP-regulated PurR-mediated activator control [5]. Purine genes are divided into at least three clusters in *L. lactis*, according to regulatory mechanisms. A small regulon comprised of *guaA* and *guaB* responds to changes in the G-nucleotide concentration by an unknown mechanism (Mogens Kilstrup, unpublished). The *guaAB* genes show only minor changes in their temporal expression patterns (Fig. 5B). In other words, the intracellular G-nucleotide concentration did not severely change during the fermentation. The *xpt-pbuX* operon is subject to riboswitch control, with the purine bases guanine or hypoxanthine as low molecular weight effectors [5], [42]. The temporal expression of these genes followed that of the PurR regulon members, suggesting that an inverse correlation exists between the levels of PRPP and free purine bases during fermentation.

It appears that the highest expression of the PurR regulon genes during milk fermentation coincides with a decrease in the growth rate. Previously, it was observed that growth in a synthetic amino-acid-supplemented SA medium resulted in a partial purine deficiency [43]. Purine addition increased the growth rate and lowered the expression of PurR regulon members. It appears that the purine deficiency, as indicated by the increased expression observed for purR genes, is due to an inability of the *de novo* pathway to meet the high consumption rate during the stressful transition into the stationary phase. Since the PurR regulon genes are induced under these conditions, the precursor PRPP pool was elevated compared to the normal level. Upon the subsequent lowering of the growth rate in the stationary phase, the consumption rate lowered and matched the production rate. This resulted in renormalization of the PRPP pool and down regulation of PurR regulon members. Analysis of the upstream DNA regions of the PurR regulon genes and genes with correlated transcription profiles is described below.

Fig. 5C shows the temporal expression patterns of the genes of the regulon of PyrR, an RNA binding protein responding to high uridine 5-monophaspate (UMP) concentrations. The PyrR regulon has one critical time point at around T3. From T1 to T3, expression of all regulon members continually increases. A subsequent decrease in expression levels until T9 is indicative of an increase in the UMP concentration. PyrR is a genuine member of the PyrR regulon and is transcribed from the PyrR-regulated *pyrRPB-carA* operon. Accordingly, *pyrR* mRNA levels follow the same trend as those of the other PyrR regulon members.

### The heat stress response genes behave as an explicit group

The temporal expression profiles of the major stress response genes *groEL2*, *groES*, *hrcA*, *dnaJ* and *dnaK* show a high correlation during growth in milk. This similar expression behavior allowed to pinpoint the common CIRCE DNA motif, see below. When *L. lactis* MG1363 was inoculated, the stress genes showed relatively low levels of expression up to T6 (T5 for *hrcA*). The expression of all genes increased when the cells entered the transition state of growth (T6 to T7). Subsequently, the expression increased even more in the early stationary growth phase (T8 to T9). There appears to be no correlation between the expression profiles of the heat stress genes and global gene expression during the transition from the early to the late stationary phase of growth. In other words, although some processes are affected by stress gene responses, the majority of processes are not correlated to the expression of stress response genes during stationary phase.

### DNA-motifs discovered on basis of gene expression profiles

Transcriptional regulators play central roles in the expression of genes by binding to specific binding sites (TFBSs) in upstream intergenic regions. To uncover DNA-motifs that are potential TFBSs, we searched for overrepresented DNA sequences in the upstream intergenic region of genes that showed correlated expression. The results are presented in the supplementary Table S1 which include a column indicating if the DNA motif is identified in the upstream region of a core genome gene of *L. lactis* [44].

A total of 58 clusters of genes identified by *k*-means clustering (using Euclidian distance) were examined using the DISCLOSE software package [30]. Many of the identified DNA-motifs that consisted of four to five bases were discarded because of their abundance. Non-palindromic elements were only selected if their general occurrence in intergenic regions of the *L. lactis* MG1363 genome was less than 5%. This manual curation pinpointed 25 new DNA-motifs as being potential motifs of regulatory significance. For each motif a weight matrix was built and used to screen all the intergenic regions in the *L. lactis* MG1363 genome for further occurrences.

The upstream regions of the genes in some clusters contained more than one potential DNA-motif whereas others contained no obvious DNA-motif. DISCLOSE identified two motifs in cluster 25, containing 11 genes. One DNA motif, GACAAAWWTWTTTGAC (motif name = llmg_m001), is located upstream of *llmg_2249*, *nifJ*, and *cadA*, which encode proteins with significant similarity to an iron-dependent oxidoreductase, a pyruvate-flavodoxin oxidoreductase, and an ion efflux ATPase, respectively. Expression of these three genes is known to be reduced in the absence of cysteine [45]. Allowing for one mismatch, similar motifs are also present upstream of the genes *llmg_1857* and *llmg_2516/llmg_2517*, that are not known to be involved in the regulon mentioned above. The second motif (TCAGTWWACTG; llmg_m002) in cluster 25 is located upstream of *pyrR* and *pyrB* and is also present upstream of *arcD1* and *acmC*. Cluster 47 also contained two motifs (llmg_m003 and llmg_m004), of which the latter (WWWCCGAACWWW) occurs 9 times and resembles the known PurBox (AWWWCCGAACWWT) [46]. It is present upstream of the purine biosynthesis genes *glyA*, *fhs*, *purC*, *purM*, *purD*, *purH* and *purR*. The other is the DNA-motif (llmg_m003) that is highly specific for purine biosynthesis; it was found upstream of only three other genes, *yajC*, *llmg_1234* and *llmg_1550* previously unlinked to purine biosynthesis. Strikingly, a tiled motif is present upstream of genes coding for other proteins involved in purine biosynthesis, namely *purL, pbuO, purM, purC*. This motif (TTCNNNNNNNNCGAAC; llmg_m003), was present upstream of seven genes unrelated to purine biosynthesis and has recently been described by Jendresen and co-workers [47].

The motif (AGCACTCWWWTWWWWAGAGTGCTAAWW; llmg_m005) is present upstream of *groES*, and *dnaK*, two member of cluster 2. This motif resembles the so-called CIRCE box; (TTAGCACT-10N-GAGTGCTAA; [48]). This box is also present upstream of *groEL2*, *hrcA* and *dnaJ*. A survey of the intergenic regions with motif llmg_m005 also identified it upstream of two other stress response genes, *hrcA* and *dnaJ*. A weight matrix allowed identifying two more copies of this sequence namely upstream of *llmg_0268* - *llmg_0269*, encoding a putative ABC-type multidrug transporter, and upstream of *sugE*, specifying a protein putatively involved in multidrug resistance.

The divergently transcribed genes *llmg_1235* and *llmg_1236*, both part of cluster 39, share a perfect palindromic sequence (TATATGAGATATCTCATATA) in their upstream region. The *llmg_1236* gene, encoding a transmembrane protein with unknown function, starts 93 bp downstream of this DNA motif while the *llmg_1235* gene, encoding for a cAMP-binding protein, is only 31 bp downstream of the same DNA motif.

Genes of cysteine metabolism (GO:0006534) are present in cluster 11, which means that their expression profiles are very similar over the complete time-series. A motif (GACWNAATATTYYGTCA; llmg_m017) in the upstream regions of three out of four genes is also present upstream of the genes of two putative ABC transporters (*llmg_1973 and llmg_0341*) and two putative membrane proteins (*llmg_1683 and llmg_2454*), all of which have no known functions.

General stress protein genes are overrepresented in cluster 49 according to the FIVA analysis but no common motif could be identified in their upstream sequences, even though the expression profiles correlated really well in this cluster.

Two genes that are possible members of the KEGG pathway “*Starch and Sucrose Metabolism*” of *L. lactis* MG1363 (path_llm00500) share the motif GCGNNNCGCT. One of them, *llmg_1195* is known to be a member of the CcpA regulon [11].

Two DNA-motifs (GAT-N9-CCC; llmg_m024 and CCC-N9-GGC; llmg_m025) are present upstream of seven and four operons, respectively, of the ten operons of cluster 32. Subsequent mapping on the genome revealed that the motifs overlap at one position: the elongated DNA motif GAT-N9-CCC-N9-GGC (llmg_m044) is present upstream of the operon *dexA, maa, amyY, agl, mapA*.

The motifs described above all contain a spacing of around 9–10 bases. This is not the case for the DNA-motif upstream of the genes in cluster 20. In this cluster, genes coding for proteins involved in the “*Organic Acid Metabolic Process*” (GO_0006082) are overrepresented. The DNA motif consists of a direct repeat with variable spacing (see Table 2). This extra freedom in spacing reduces the significance of the score for this kind of motif, which is not easily discovered by the current DNA motif mining tools. This would suggest that many more interesting DNA motifs still remain to be discovered.

**Table 2:** Motifs with variable spacing between the conserved DNA repeats present upstream of the genes in cluster 20.

*lysS (llmg_0389)*	TGTCAGT 5N TGTaAGT
*lysA (llmg_1185)*	TGTCAGT 11N TGTCAGT
*leuC (llmg_1281)*	TGTCAGT 39N TGTCAGT
*hom (llmg_1331)*	TGTCAGT 32N TGTCAGT
*arcD1 (llmg_2311)*	TGTCAGT 11N TGTCAGT
*llmg_2586*	TGTCAGT 22N TGTCAGT

### Discovery of DNA-motifs based on known regulons

The method used to discover regulatory motifs was also applied to genes belonging to the same regulon. In this way, 10 additional motifs were identified.

The expression of arginine metabolism in *L. lactis* is controlled by the two homologous transcriptional regulators ArgR and AhrC. Using the upstream DNA regions of genes under control of AhrC, the obtained DNA motif (GTATWATWATA; llmg_m027) is comparable with the AhrC DNA binding motif known from literature (RNATAWWWRWRCW [49]). A weight matrix search using motif llmg_m027 identified this motif upstream of all AhrC members except the operon argGH. No significant motif could be obtained for the ArgR regulon because the known ArgR motif (Larsen et al) is very AT rich leading to a high background.

Interestingly, next to the known PyR DNA motif (ARTCCNGNGAGGYT, [50]), an overrepresented DNA motif (CTGACAGWTCTRTCA ; llmg_m043) is present in the upstream regions of all PyR regulon members.

The transcription profiles of the homologous transcriptional regulator genes *flpA* and *flpB* show a high correlation and we could discern a highly conserved motif (TTTAWRMWYMGWWYMGCGG ; llmg_m028) that is specific for these two genes. It is located at −200 relative to the start codon of each gene. Although a putative DNA binding motif has been proposed for FlpA of *L. lactis* IL1403 [51], this sequence is not present in the upstream region of *flpB* in *L. lactis* strains IL1403 and MG1363.

Analysis of the regulon of the global regulator CodY revealed that besides the known CodY-Box [12] a second motif is present. In three of the CodY-regulated operons this motif is not located in the upstream intergenic regions but in the relatively large intergenic region between the second and the third gene of each operon. It is not clear whether this motif at such an unusual location could have any function.

*L. lactis* CcpA is a global regulator controlling the expression of a large number of genes [52]. The expression profiles of several CcpA regulon members differ during growth in milk, suggesting that other regulators are also involved in the expression of these genes. Indeed, in addition to the known CcpA DNA-binding motif *cre*, two additional motifs (llmg_m032, llmg_m033) in the upstream regions of several CcpA regulon members could be involved in their fine tuning.

In total 36 new DNA-motifs were identified in the genome of *L. lactis* MG1363, of which 29 were pinpointed on the basis of the clustering results and 10 were identified using regulon classification data. To make this valuable information easily accessible, a database was made from the 27 known and the 39 new DNA-motifs, the consensus sequences of which were built from the alignments (see Supplementary Materials, Table S1 and http://milkts.molgenrug.nl). Furthermore, the database contains all DNA sequence elements from which the motifs were derived.

### Building an *L. lactis* GRN

**Figure 6:**
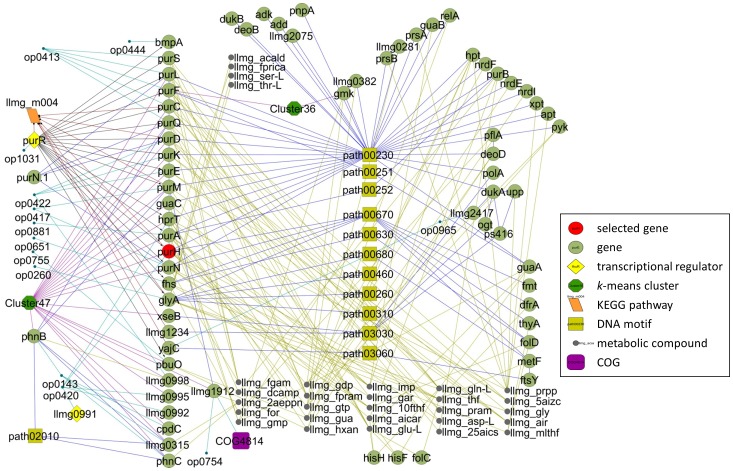
Gene Network Reconstruction of *L. lactis* milk fermentation; *purH* example. First and second order connections to *purH* are visualized in Cytoscape [52]. The *purH* gene is marked in red. For further explanation of the symbols (nodes) see inset. For pathway description see Table 4. Relations between nodes are shown as lines; blue: GeneNet and pathways; purple: *k*-means clustering; black: regulon; red: DNA motifs; green: compounds and light blue: operons.

Using the “Network Building Blocks” presented Table 3 a Gene Regulatory Network (GRN) was built and visualized in Cytoscape [53] (Fig. 6). The GRN obtained in this way is especially useful for discovery of interactions between genes, regulatory elements and metabolic compounds. One straightforward method to mine the GRN is to build the network step by step, starting with a single gene or operon of interest and subsequently adding coupled information, such as regulons, TFBSs, gene-to-gene expression correlation and metabolic compounds and pathways. Subsequently, these connected items (first order connection) can be used to expand the network to a second order.

**Table 3:** Gene Network building blocks used to reconstruct the GRN operative in *L. lactis* MG1363 during milk fermentation.

Block #	Building block name	Description	# Nodes	# Edges
1	Expression	Gene expression of all genes over time	2516	0
2	Clusters_*k*-means	*k*-means clustering on signal data using 58 clusters and Euclidian distance	709	588
3	GeneNet099	Correlation between genes derived from GeneNet with correlation *p*-value>0.99	1010	11867
4	GeneNet095	Correlation between genes derived from GeneNet correlation *p*-value>0.95		
5	Pearson	Pearson's correlation between genes *p*-value>0.95	1264	5882
6	Spearman	Spearman correlation between genes *p*-value>0.95		
7	Motifs	Interaction between gene and its upstream DNA motif. New motifs, as described in this article, and literature-based motifs	190	182
8	Operons	Describes interaction between genes when they are members of the same operon. Prediction is calculated as described in this article	3039	2432
9	Compound	Production and consumption of compounds by the product of a gene	996	1526
10	Regulon	Positive or negative interaction between regulator and genes	187	204
11	Pathways	Interaction based on KEGG metabolic pathways	994	1253
12	Node_description	Describes gene classification	1967	0
13	VizProp	Describes Node and Edge shape and colour		

**Table 4:** Description of the KEGG pathways presented in Fig. 6.

KEGG pathway	Description
path00230	Purine metabolism
path00251	Glutamate metabolism
path00252	Alanine and aspartate metabolism
path00260	Glycine, serine and threonine metabolism
path00310	Lysine degradation
path00460	Cyanoamino acid metabolism
path00630	Glyoxylate and dicarboxylate metabolism
path00670	One carbon pool by folate
path00680	Methane metabolism
path03030	DNA polymerase
path03060	Protein export

As an example we present the network around the bi-functional purine biosynthesis protein PurH (EC 2.1.2.3; Phosphoribosylaminoimidazolecarboxamide formyltransferase and EC3.5.4.10; IMP cyclohydrolase). All first neighbors of *purH* (first order connection) and all neighbors of these neighbors (second order connection) were selected. The resulting network links (i) all genes having the same expression profile as *purH*, (ii) the members of the PurR-regulon, of which *purH* is one, (iii) the operon members, (iv) metabolic pathways of which PurH is a member, (v) all compounds produced and consumed by PurH and (vi) the genes having the same TFBS as purH has in their upstream regions. Investigating the genomic context, the first gene downstream of *purH*, *llmg_0995*, encoding a putative hydrolases of the HAD superfamily, is linked to *purH* via Cluster47 (*k*-means clustering results), meaning that their expression profiles are highly similar. The gene *llmg_0996* is transcribed in the opposite direction to *purH* and *llmg_0995* and is not correlated to the two with respect to gene expression. The genes *purE*, *purK*, *purD* and *llmg_0998* of operon *op0422* have a second-order interaction with *purH* indicating that their expression profiles have a low correlation to that of *purH*, especially in the stationary phase the expression levels of *purE* and *purK* are much lower than those of *purH* and *llmg_0995*. The operon (*op0417*) containing *purM* and *purN*, encoding two of the eleven enzymes required for the fifth step in purine biosynthesis, has first-order gene expression connection to *purH*. The transcriptional network shows that DNA motif llmg_m004 is upstream of *glyA*, *purR*, *yajC*, *llmg1234*, *purD*, *purM*, *purH* and *purC*. Genes downstream of *purC* and in the same operon (*purCSQLF*) are also linked to llmg_m004, which is revealed by the GRN.

In the next step of GRN building data of transcription regulation derived from literature was added, extending the network with genes under control of PurR. The PurBox's described by Kilstrup and Jendresen [44], [45] was not included in this network because it defines a poorly conserved motif and would add many irrelevant genes to the GRN. Subsequently, integration of the *k*-means clusters derived from DISCLOSE adds links between genes with similar expression profiles over the complete time-series. Cluster 47 contains the majority of the pur genes (operons: *purCSQLF, purDEK llmg0998, purMN, purH*) but the GRN also links a putative phosphonate transporter operon (*phnB phnC llmg_0315 cpdC*), and the guanine permease gene *pbuO*, Furthermore, several genes encoding hypothetical proteins are now linked to known genes on the basis of their expression profiles and other edges of the transcriptional network. Thus *llmg_1912* is linked to *pnuO*, *llmg_0998* to *purDEK*, *llmg_0995* to *purH* and *llmg_315* to *phnBC*. Interestingly, *glyA* and *purD* are involved in consumption of compound llmg_gly while they also have the same upstream motif llmg_m004.

This single example shows the power of GRNs to discover links between various biological systems. It is beyond the scope of this work to describe all the interesting findings derived from this GRN. The network built here, starting with *purH* as the centre node, is available as an XGML file (“subnetwork_purH.xgmml”) for Cytoscape. The complete network can be downloaded from our website.

## Conclusion

This analysis shows how genes and classes of genes behave when *L. lactis* is grown in milk. Deep insights in the processes of gene regulation are gained and leads for future experimental research can be obtained from this detailed analysis of this milk transcriptome time-series. As an example, we uncovered a substantial number of novel putative TFBSs which seem to be operational during growth in milk. Finally, all results were used to build the *L. lactis* milk fermentation GRN. The GRN building blocks that we used are freely available on-line and will give researchers in the field the exiting possibility to mine and visualize in detail their process of interest. In another paper this valuable resource is extended with an in-depth elucidation of the growth behavior of *L. lactis* MG1363 in rich GM17 medium.

## Supporting Information

Table S1 **An overview of the identified DNA motifs on the basis of clustering or regulon data.**
(XLSX)
